# Identification of differentially expressed genes between sorghum genotypes with contrasting nitrogen stress tolerance by genome-wide transcriptional profiling

**DOI:** 10.1186/1471-2164-15-179

**Published:** 2014-03-05

**Authors:** Malleswari Gelli, Yongchao Duo, Anji Reddy Konda, Chi Zhang, David Holding, Ismail Dweikat

**Affiliations:** 1 Department of Agronomy and Horticulture, University of Nebraska, Lincoln, NE 68588, USA; 2 School of Biological Sciences, University of Nebraska, Lincoln, NE 68588, USA; 3 Center for Plant Science Innovation, University of Nebraska, Lincoln, NE 68588, USA; 4 Department of Biochemistry, University of Nebraska, Lincoln, NE 68588, USA

**Keywords:** N-stress, Sorghum, Nitrogen use efficiency, Transcriptome, RNA-seq, Genotypes, Differentially expressed genes

## Abstract

**Background:**

Sorghum is an important cereal crop, which requires large quantities of nitrogen fertilizer for achieving commercial yields. Identification of the genes responsible for low-N tolerance in sorghum will facilitate understanding of the molecular mechanisms of low-N tolerance, and also facilitate the genetic improvement of sorghum through marker-assisted selection or gene transformation. In this study we compared the transcriptomes of root tissues from seven sorghum genotypes having differential response to low-N stress.

**Results:**

Illumina RNA-sequencing detected several common differentially expressed genes (DEGs) between four low-N tolerant sorghum genotypes (San Chi San, China17, KS78 and high-NUE bulk) and three sensitive genotypes (CK60, BTx623 and low-NUE bulk). In sensitive genotypes, N-stress increased the abundance of DEG transcripts associated with stress responses including oxidative stress and stimuli were abundant. The tolerant genotypes adapt to N deficiency by producing greater root mass for efficient uptake of nutrients. In tolerant genotypes, higher abundance of transcripts related to high affinity nitrate transporters (NRT2.2, NRT2.3, NRT2.5, and NRT2.6) and lysine histidine transporter 1 (LHT1), may suggest an improved uptake efficiency of inorganic and organic forms of nitrogen. Higher abundance of SEC14 cytosolic factor family protein transcript in tolerant genotypes could lead to increased membrane stability and tolerance to N-stress.

**Conclusions:**

Comparison of transcriptomes between N-stress tolerant and sensitive genotypes revealed several common DEG transcripts. Some of these DEGs were evaluated further by comparing the transcriptomes of genotypes grown under full N. The DEG transcripts showed higher expression in tolerant genotypes could be used for transgenic over-expression in sensitive genotypes of sorghum and related crops for increased tolerance to N-stress, which results in increased nitrogen use efficiency for sustainable agriculture.

## Background

Sorghum [*Sorghum bicolor* (L.) Moench] is one of the most important staple food grain crops for millions of people living in the West Africa and India [1]. Sorghum performs C_4_ photosynthesis, which makes it adapted to high temperatures and water limitation [2]. Despite its C_4_ nature, sorghum depends on nitrogen fertilizers for high grain yields. In higher plants, N limitation leads to dramatic changes in plant growth and development, such as root branching, leaf chlorosis and reduced seed production [3,4]. Nitrogen is a constituent of amino acids, nucleotides, proteins, chlorophyll, and several plant hormones. It is an important inorganic nutrient for plant growth and development [5,6].

Nitrate is the major source of N in agricultural soils [7], serving both as a nutrient and a signal [3]. As a nutrient, it is absorbed by roots through low- and high-affinity nitrate transporters (NRT1 and NRT2), which is reduced to nitrite by nitrate reductase (NR), and to ammonium by nitrite reductase (NiR). Ammonium is then incorporated into amino acids by glutamine synthetase (GS) and glutamate synthase (GOGAT) [8,3,9]. Localized supply of nitrate strongly promotes the elongation of lateral roots [5]. As a signal, nitrate induces the expression of a number of genes including NRT1, NRT2, NR and NiR [3,10], GS and GOGAT [3,9]. In addition to these nitrogen metabolism genes, expression of different regulatory genes also induced by nitrate. For example, nitrate stimulates the expression of the *Arabidopsis* MADS-box gene, ANR1, regulates lateral root development [5]. It also induces AFG3 (Auxin signaling F-box 3) and which enhances miR393 levels to modulate root architecture [11].

In the past several decades, the increasing use of nitrogen fertilizers in crop production has played a major role in improving yields [6], which underlies our current population growth. However, crop plants use less than half of the applied nitrogen [12]. Excess nitrate volatilizes as reactive N gases by denitrifying bacteria [13] or leaches into waterways and causes eutrophication. Recent analysis showed that acidification of soil results mainly from high usage of N fertilizers [14]. The heavy reliance on fertilizer application has resulted in greater need for environmental protection measures. Therefore, improving nitrogen use efficiency (NUE) by developing genotypes that yield better with limited N supply is a prerequisite for sustainable agriculture. NUE is defined as the amount of biomass and grain yield produced per unit of available N in the soil [15]. The molecular basis of the NUE traits is complex. Genetic variation exists for NUE in sorghum [16] and maize [17], suggesting that scope exists for selecting high NUE genotypes. Interestingly, comparison of N uptake capacities of maize and sorghum under contrasting levels of N availability showed that under non-limiting N supply, the two crops have similar N uptake, while under severe N-limitation the N uptake capacity of sorghum is higher than that of maize [18]. The reason for this difference is unclear, but it could be due to a more developed and branched root system in sorghum compared to maize. Hirel et al. [19] suggested the components involved in N uptake capacity of sorghum are potential candidates for improving N uptake capacity of maize and possibly other crops under N-limiting conditions.

Many efforts have been made to understand the molecular basis of plant responses to N and identifying N-responsive genes in order to manipulate their expression and enable plants to use N more efficiently [20]. In *Arabidopsis*, microarray analysis of gene expression changes in response to different concentrations of nitrate for both short-term and long-term treatments revealed numerous genes involved in nitrogen response [21,22]. In rice, Lian et al. [23] reported expression profiles of 10,422 unique genes using a microarray, while no significant difference was detected in the transcriptomes of leaf tissues, and a total of 471 genes showed differential expression in the root tissues in response to low-N stress. Bi et al. [24] developed a growth system for rice by limiting N and identified N-responsive genes, validated the function of an early nodulin gene, *OsENOD93-1*, by over-expressing in rice. Some of these experiments were performed with a short period of N-stress and identified differentially expressed genes in response to the N-stress in *Arabidopsis*[21] and rice [23]. A transcriptional change in response to longer periods of stress, which is crucial for adaptation to field conditions, has also been identified [22,24]. However, a limitation in these experiments was the use of single genotype. Without comparing the transcriptional differences between N-stress tolerant and sensitive genotypes, it is impossible to separate N-stress tolerant genes from stress responsive genes.

In maize, Chen et al. [25] detected many nitrogen responsive genes by analyzing the global gene expression changes in response to N-stress in leaf tissues of two maize inbred lines with contrasting N-stress tolerance using an affymetrix maize genome array. The transcriptional profiling of two soybean genotypes exposed to N-stress using Illumina RNA-sequencing revealed a number of candidate genes for N utilization [26]. Investigating the N-stress tolerance mechanisms in sorghum could facilitate a better understanding of the genetic bases of low-N tolerance, and so enable the effective use of genetic and genomic approaches to improve sorghum N-stress tolerance. To identify the genes responsible for stress tolerance, genotypes with similar genetic backgrounds, but with contrasting stress tolerance, are ideal for linking candidate genes to the stress tolerance. However, developing such near-isogenic lines requires several years of backcrossing and selection [27]. One alternative is to identify common genes that are differentially expressed between low-N tolerant and sensitive genotypes with different genetic backgrounds under N-stress conditions.

To this end, we conducted transcriptional profiling of seven sorghum genotypes (four low-N tolerant and three low-N sensitive) having differential phenotypic response to N-stress using RNA-seq technology. In this case, we maximized the number of lines analyzed in an attempt to identify common differentially expressed genes (DEGs). We identified a number of common N-stress tolerant DEGs between sensitive and tolerant genotypes under N-limited conditions.

## Methods

### Generating plant material and screening for N-stress tolerance under field conditions

The physiological adaptations to N-stress were compared between two Chinese sorghum lines (China17 and San Chi San) with two U.S. sorghum lines, CK60 and BTx623 grown in greenhouse conditions. The biochemical assays conducted on these genotypes by Maranville and Madhavan [28] showed that assimilation efficiency index and phosphoenolpyruvate carboxylase (PEPcase) activity were significantly greater for the Chinese lines than the U.S. lines. In this project, we developed 210 F_7_ Recombinant Inbred Lines (RILs) by crossing the low-N sensitive U.S line, CK60 with the day-length insensitive and low-N tolerant Chinese line, San Chi San. Each of the RILs was derived from a single F_2_ plant following the single seed descent method until F_7_ generation. Sorghum genotypes KS78, BTx623, CK60, San Chi San, China17 and the F_7_ RILs were evaluated phenotypically in two N regimes for two years with two replications each. Field experiments were conducted at University of Nebraska-Lincoln experimental farms at Mead, Nebraska and consisted of low-N (LN, 0 kg ha^-1^) and normal N (NN, 100 kg ha^-1^) regimes. The LN field had not received any applied nitrogen fertilizer since 1986. Plant height (PH) was measured from base of the plant to tip of the head in centimeter. Biomass and grain yields (BY and GY, t ha^-1^) were recorded under both N regimes. Five of the worst performing RILs (RILs 1-5) and five of the best performing RILs (RILs 6-10) covering the two tails of CK60 × San Chi San population were selected based on their biomass yield (t ha^-1^) under LN conditions.

### Screening the selected genotypes for N-stress under controlled conditions

Seeds from KS78, BTx623, CK60, San Chi San, and China17 sorghum genotypes, five best and worst performing RILs selected from LN field conditions, were planted in Sunshine mix (Canadian sphagnum peat moss, vermiculite, and dolomitic limestone) without added fertilizer (N-stress). These genotypes were also planted in Sunshine mix provided with 100% Hoagland solution (Full N) [29]. The seeds were grown in three inch pots under a 16/8 h photoperiod at 25°C (day) and 18°C (night). The fresh and dry weights of root and shoot tissues of three week old seedlings were measured from both N-conditions.

### RNA extraction from root tissues

The roots were harvested separately from three week old seedlings, all traces of soil removed by repeated gentle washing in de-ionized water, frozen in liquid nitrogen and stored at -80°C until RNA extraction. All samples were taken at middle of the day to minimize diurnal changes in C and N metabolism [30], because the expression levels of nitrate assimilation genes are different at different time points of the day. Total RNA was extracted first using NTES buffer (20 mM TRIS pH 8, 10 mM EDTA, 100 mM NaCl and 1% SDS) and followed by Trizol reagent (Invitrogen) using the manufacturer’s instructions. RNA samples were dissolved in RNAse-free H_2_O, the integrity and quality of the total RNA was checked by a NanoDrop 1000 spectrophotometer and by resolution on a 1% non-denaturing agarose gels. Equal quantities of RNA from the five best performing RILs and the five worst performing RILs were bulked as high-NUE and low-NUE bulks respectively. For RNA-seq, four biological replications of each genotype grown under N-stress were used.

### Illumina RNA-sequencing

RNA-seq was used to identify common DEG transcripts among root tissues of four N-stress tolerant genotypes (San Chi San, China17, KS78, and the high-NUE bulk) and three sensitive genotypes [CK60, BTx623 (reference genome), and low-NUE bulk] grown under N-stress. The experimental process is summarized as follows: RNA libraries were prepared from 4 μg total RNA using the Illumina TruSeq RNA Sample Prep Kit v2 - Set A (RS-122-2002) according to the manufacturer’s instructions. Libraries were analyzed and measured by gel electrophoresis and NanoDrop 1000 Spectrophotometer to a concentration of 10 nM each. Four indexed libraries were pooled into one lane and clusters generated at 8 pM concentration were sequenced on the Illumina Genome Analyzer IIx (GAIIx; Illumina, Inc., San Diego, CA) using three 36-cycle sequencing kits to read 76 nucleotides of sequence from a single end of each insert, by standard multiplexing v8.3 protocol.

### Identification of Differentially Expressed Genes

Short reads with 76 bp generated by GAIIx were initially processed to remove the adapter sequences and low-quality bases at the 3’ end. The short reads were mapped against the *Sorghum bicolor* 79 genome (http://www.phytozome.net/sorghum.php) using Bowtie [31], allowing up to two mismatches. The reads mapped to multiple locations were discarded. The number of reads in genes was counted by HTSeq-count tool [32] with the ‘union’ resolution mode. Then, the edgeR package [33] with TMM normalization method was used to align expression values to a common scale. The reads per kilo base per million (RPKM) values were also calculated for genes as the expression level [34]. The resulting expression values were log_2_-transformed. Average log signal values of four biological replications for each sample were then computed and used for further analysis. The cutoff of log_2_-fold value ≥1 (2-fold absolute value) and adjusted P-value <0.001 (FDR) were used for determining significant DEG transcripts. A total of 12 pair-wise comparisons were made by comparing three sensitive genotypes with each of the four tolerant genotypes to find common DEG transcripts across all genotypes. In addition, tolerant and sensitive genotypes were compared one by one to each other among themselves to asses if the differences in gene expression between sensitive and tolerant genotypes found are usual or unusual for differences among sorghum genotypes.

### Gene Ontology analysis

Sorghum gene ontology (GO) term association information was obtained from http://www.phytozome.net. Using the above gene association file and the GO ID to term index file, the GO annotation file for sorghum was generated by a custom script. The GO::TermFinder [35] was used for enrichment analysis. The GO term with P ≤ 0.05 is defined as enriched GO term with significant DEGs among 12 pair-wise comparisons. This analysis allowed us to determine the major biological functions of DEGs.

### Pathway enrichment analysis

The gene pathway mappings were downloaded from http://genome.jgi-psf.org/Sorbi1/ Sorbi1.download. ftp.html and the filtered model 6 was used in the analysis. The hypergeometric test was applied to identify significantly enriched pathways:

$$P=1-\sum_{i=0}^{m=1}\frac{\left(\frac{M}{i}\right)\;\left(\frac{N-M}{n-i}\right)}{\left(\frac{N}{n}\right)}$$

Where *N* is the number of all genes with pathway annotation, *n* is the number of DEGs in *N*, *M* is the number of genes mapped to a given pathway, and *m* is the number of DEGs in *M*.

The pathways with a P value of ≤ 0.05 are defined as those with significantly enriched genes among 12 pair-wise comparisons.

### Real-time quantitative RT-PCR (qRT-PCR) analysis

qRT-PCR was used to validate and compare the expression of DEG transcripts obtained from RNA-seq experiment on the cDNA synthesized from root tissues grown under N-stress as well as full N. DEG transcripts were analyzed through qRT-PCR using an iQ™5 optical system (Bio Rad, Hercules, CA). Template cDNA samples were prepared using the iScript First Strand Synthesis System Kit (Bio-Rad) for reverse transcriptase-PCR with 500 ng of total RNA. Primers for the PCR reactions were designed to have a melting temperature of 58°C to 62°C and to produce a PCR product between 100 to 150 bp. Six differentially expressed genes were selected to validate the RNA-seq data using qRT-PCR on independent biological replicates. Primers were listed in Additional file 1. The control gene, actin (Sb01g010030) was selected since its expression was found to be stable between the root RNA extracted from different genotypes. Transcript abundance was assayed using SYBR green PCR master mix with 2 μl of 10-fold diluted cDNA and 2 μl of the primers (5 μM). The program used was as follows: initial denaturation for 3 min at 95°C, followed by 40 PCR cycles consisting of 95°C for 10 s, 56°C to 62°C for 30 s, 95°C for 60 s and 55°C for 10 s. For each product, the threshold cycle (CT), where the amplification reaction enters the exponential phase, was determined for three technical replicates and three independent biological replicates per genotype. The comparative 2^–ΔΔCT^ method was used to quantify the relative abundance of transcripts [36].

## Results

### Phenotypic performance of sorghum genotypes under field and controlled conditions

Mean phenotypic performance of the five sorghum genotypes CK60, BTx623, KS78, San Chi San and China17, and the five worst and best performing CK60 × San Chi San RILs tested under NN and LN field conditions were shown in Table 1. Under LN, the biomass and grain yield of sensitive genotypes, CK60 (3.1 t ha^-1^ and 1.1 t ha^-1^) and BTx623 (4 t ha^-1^ and 1.2 t ha^-1^), were lower than the tolerant genotypes KS78 (5.9 t ha^-1^ and 2.2 t ha^-1^), San Chi San (7.6 t ha^-1^ and 5.0 t ha^-1^) and China17 (7.3 t ha^-1^ and 3.9 t ha^-1^), respectively. The biomass and grain yields of RILs 1-5 range from 3 to 3.7 t ha^-1^ and 0.9 to 1.7 t ha^-1^ respectively, which were close to the sensitive genotypes. The biomass and grain yields of RILs 6-10 range from 9.4 to 16.5 t ha^-1^ and 1.0 to 6.7 t ha^-1^ respectively and were higher than the biomass and grain yield of LN tolerant genotypes.

**Table 1:** Performance of five sorghum inbreds, five worst (1-5) and five best performing (6-10) CK60 × San Chi San RILs grown under normal-N and low-N field conditions

**Genotype**	**Plant height (cm)**		**Biomass yield (t ha**^**-1**^**)**		**Grain yield (t ha**^**-1**^**)**	
	**NN**	**LN**	**NN**	**LN**	**NN**	**LN**
RIL-1	113	95	4.1	3.0	1.3	0.9
RIL-2	132	83	9.0	3.1	3.1	1.4
RIL-3	149	77	7.5	3.2	1.6	1.1
RIL-4	147	98	7.6	3.5	3.2	1.7
RIL-5	122	109	8.1	3.7	2.5	0.9
CK60	115	91	6.6	3.1	2.9	1.1
BTx623	140	126	8.2	4.0	2.8	1.2
KS78	132	76	10.1	5.9	4.1	2.2
San Chi San	157	137	16.5	7.6	6.4	5.0
China-17	170	157	13.8	7.3	5.5	3.9
RIL-6	124	93	13.0	11.4	6.2	4.6
RIL-7	152	122	13.2	9.6	4.0	1.0
RIL-8	161	125	13.4	9.4	3.4	2.6
RIL-9	185	168	17.7	15.1	6.2	5.2
RIL-10	163	137	18.4	16.5	7.7	6.7

NN- normal N field (100 kg ha^-1^ fertilizer); LN- low N field (0 kg ha^-1^ fertilizer).

Root systems from N-stress tolerant genotypes were usually more extensive than those of N-sensitive genotypes (not shown). To quantify these differences, we compared root biomass of all genotypes grown under no added N and full-N conditions. Selected genotypes from field evaluations were grown for three weeks in Sunshine mix provided with 100% Hoagland solution (full N) and provided with no added fertilizer (N-stress). The fresh and dry weights of root and shoot tissues from five seedlings were averaged and shown in Table 2. Under N-stress, the average weights of sensitive genotypes and worst performing RILs (1-5) were lower than the tolerant genotypes and best performing RILs (6-10).

**Table 2:** Performance of three-week old seedlings of five sorghum inbreds, five worst (1-5) and five best performing (6-10) performing RILs grown under controlled conditions

	**Full N**				**N-stress**			
**Genotype**	**Fresh weight (g)**		**Dry weight (g)**		**Fresh weight (g)**		**Dry weight (g)**	
	**Root**	**Shoot**	**Root**	**Shoot**	**Root**	**Shoot**	**Root**	**Shoot**
Ril-1	10.5	20.4	0.45	1.83	6.20	3.71	0.35	0.45
Ril-2	9.51	19.0	0.49	1.81	3.71	2.25	0.16	0.21
Ril-3	12.1	21.5	0.72	1.81	6.23	3.45	0.26	0.47
Ril-4	12.2	20.1	0.65	1.64	7.2	3.16	0.35	0.48
Ril-5	9.42	19.2	0.42	1.22	5.86	3.12	0.23	0.33
CK60	10.3	17.9	0.41	1.65	5.10	3.21	0.24	0.41
BTx623	13.0	22.5	0.66	2.00	6.50	3.50	0.44	0.52
KS78	14.1	24.3	0.83	2.36	7.51	4.35	0.50	0.70
San Chi San	12.5	24.7	0.79	2.21	6.89	4.15	0.47	0.59
China17	12.6	26.6	0.77	2.65	7.23	4.49	0.51	0.63
RIL-6	10.5	24.5	0.54	2.34	5.65	3.95	0.36	0.50
RIL-7	16.4	27.7	0.87	2.89	6.85	4.41	0.38	0.53
RIL-8	10.9	23.8	0.52	2.23	5.71	3.75	0.39	0.49
RIL-9	14.6	27.8	0.67	2.44	6.66	4.85	0.41	0.65
RIL-10	13.5	23.1	0.77	2.00	8.35	4.75	0.50	0.48

Full N-full nitrogen (Sunshine mix with 100% Hoagland solution); N-stress- nitrogen stress (Sunshine mix without added fertilizer).

### RNA-seq data analysis

We sought to compare the transcriptomes of multiple N-stress tolerant and sensitive genotypes. To select a tissue type for the RNA-seq, we conducted extensive 2D proteomic comparisons on both leaf and root tissue extracts from three week old seedlings of sensitive and tolerant genotypes grown on Murashige and Skoog medium, as well as 45 day old leaves from soil grown plants, in the presence and absence of added N (not shown). In general, greater protein abundance differences were observed between the root tissues of sensitive and tolerant genotypes grown under N-stress compared to full N. In contrast, no such generalized increase in protein abundance or obvious changes in individual proteins were observed between leaf tissues of three week-old and 45 day old plants grown at either N condition (data was not shown). Therefore, in this study, we focused our transcriptional profiling experiments on root tissues.

Seeds from the selected genotypes were grown in Sunshine mix without fertilizer (N-stress) for three weeks. The seedlings of KS78, China17, San Chi San and best performing RILs have higher root and shoot mass compared to CK60 and worst performing RILs (Table 2). To survey the root transcriptome in response to N-stress, cDNA samples were prepared from the root tissues of seven sorghum genotypes [CK60 (1), BTx623 (2), San Chi San (3), China17 (4), KS78 (5), high (6) and low (7) NUE bulks] grown under N-stress conditions and used for Illumina RNA-seq. The total number of reads generated from each library (average of four biological replications) ranged from 7.3 to 8.3 million (Table 3). After filtering, the sequences of the seven libraries were mapped to the sorghum reference genome, and a total of 5851955, 5306235, 5018702, 5399517, 6182990, 5269488, 5751263 sequences were matched (~70% of the reads). The number of reads producing unique sequences ranged from 3.6 to 5.2 million. The number of genes with at least one mapped transcript was 21497, 21537, 21295, 21160, 21632, 21336 and 22033 for libraries 1, 2, 3, 4, 5, 6, and 7, respectively. Similarly, the number of genes with RPKM ≥ 1 was 14972, 14452, 14678, 13924, 14369, 15037 and 14673 for seven libraries respectively.

**Table 3:** Categorization and abundance of transcripts detected with RNA-seq

**Summary**	**Lib 1**	**Lib 2**	**Lib 3**	**Lib 4**	**Lib 5**	**Lib 6**	**Lib 7**
Total raw reads	8312677	7422797	7462817	7695810	8268795	7329570	7907043
Reads mapped to reference	5851955	5306235	5018702	5399517	6182990	5269488	5751263
Alignment	6467410	5758740	5324164	6623514	7615114	6899562	6842041
Unique mapped reads	5236500	4853730	4713240	4175521	4750866	3639414	4660485
Reads in gene region	4810502	4465738	4342745	3816552	4353451	3287048	4261630
Genes with ≥1 reads	21497	21537	21295	21160	21632	21336	22033
Gene # when RPKM is at least 1	14972	14452	14678	13924	14369	15037	14673

Lib 1-7: CK60, BTx623, San Chi San, China17, KS78, high- and low-NUE RIL bulk libraries respectively.

### Differential transcript abundance between sorghum genotypes

**Figure 1:**
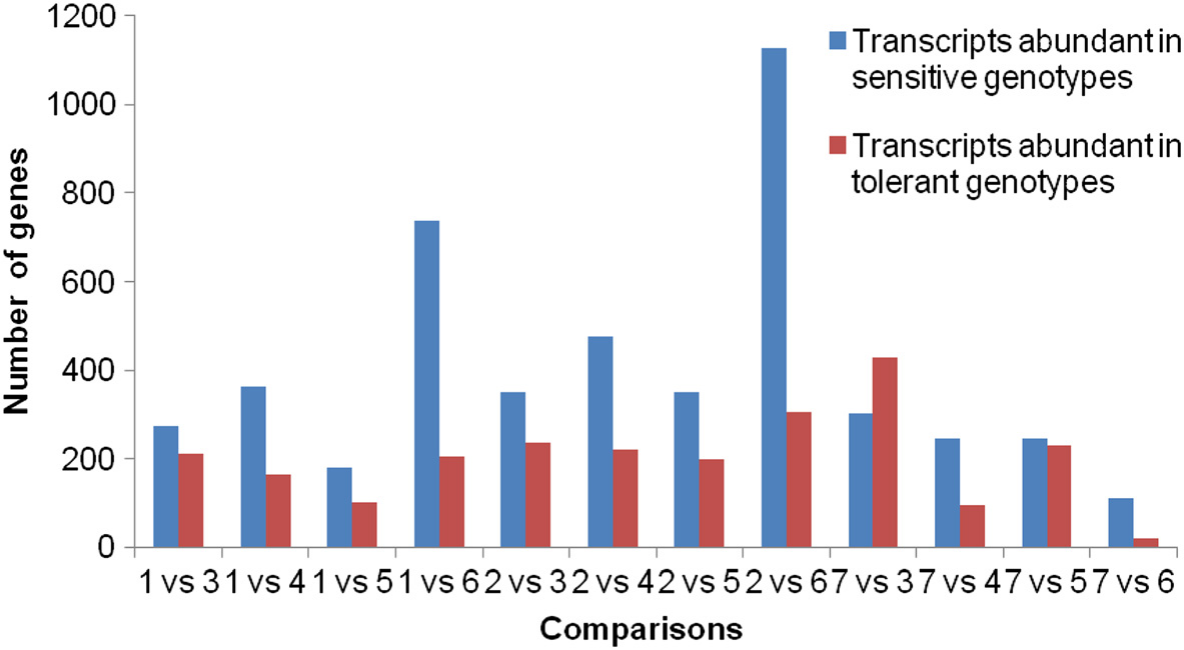
**Number of differentially expressed gene transcripts between sensitive and tolerant genotypes among seven libraries.** 12 pair-wise comparisons (1/3, 1/4, 1/5, 1/6, 2/3, 2/4, 2/5, 2/6, 7/3, 7/4, 7/5, and 7/6) were made by comparing three sensitive genotypes [CK60 (1), BTx623 (2) and the low-NUE bulk (7)] with each of the four tolerant genotypes [San Chi San (3), China17 (4), KS78 (5) and the high-NUE bulk (6)].

To check the variation in transcript abundance between low-N sensitive and tolerant genotypes, 12 pair-wise comparisons were made between three sensitive genotypes [CK60 (1), BTx623 (2) and low-NUE bulk (7)] with each of the four tolerant genotypes [San Chi San (3), China17 (4), KS78 (5) and the high-NUE bulk (6)]. A total of 486, 527, 279, 941, 589, 695, 550, 1432, 731, 341, 478, and 130 gene transcripts were found to be differentially expressed between 1/3, 1/4, 1/5, 1/6, 2/3, 2/4, 2/5, 2/6, 7/3, 7/4, 7/5, and 7/6 library comparisons (Additional file 2, Figure 1). Higher number of DEG transcripts were observed in BTx623 (1432), and CK60 (941) when compared with the high-NUE bulk (Figure 1). Four tolerant genotypes (3, 4, 5, and 6) were compared one by one to each other, 60 gene transcripts showed differential expression in at least five pair-wise comparisons (Additional file 3). Similarly, pair-wise comparisons among three sensitive genotypes (1, 2, and 7) showed 289 transcripts were differentially expressed in at least two comparisons (Additional file 4).

### Confirmation of differentially expressed candidate genes

To confirm the gene expression profiling data obtained from RNA-seq, qRT-PCR analysis was used to test the expression of selected candidate genes. The gene specific primers used are listed (Additional file 1). For the genes tested, the differential expression observed with RNA-seq was generally confirmed with qRT-PCR data (Additional file 5). Since gene expression differences could also result from responses to other deficient nutrients, we tested the expression profiles of the same selected candidate genes in root tissues grown under full N provided with 100% Hoagland solution (Additional file 5). In general, genes that were differentially expressed between sensitive and tolerant genotypes, under low-N were either not differentially expressed or had less pronounced differential expression when grown under full N conditions (Additional file 5). For example, 2OG-Fe oxygenase (Sb08g016370) which had dramatically increased expression in the N-sensitive genotypes between most pair-wise comparisons under low-N (Additional file 5C), was not increased in root tissues of sensitive genotypes when plants grown under full N (Additional file 5C). A disease resistance gene (Sb05g008910) was differentially expressed to a lesser extent in full N conditions than in N-limited conditions, in most pair-wise comparisons (Additional file 5B and C). Furthermore, an aquaporin gene (Sb10g007610), which was strongly increased in expression in most of the sensitive genotypes under N-stress, was generally decreased in expression relative to N-stress tolerant genotypes, under full-N. This is reflected by fold-change values of less than one (Additional file 5C).

### Gene Ontology functional annotation of DEGs

**Figure 2:**
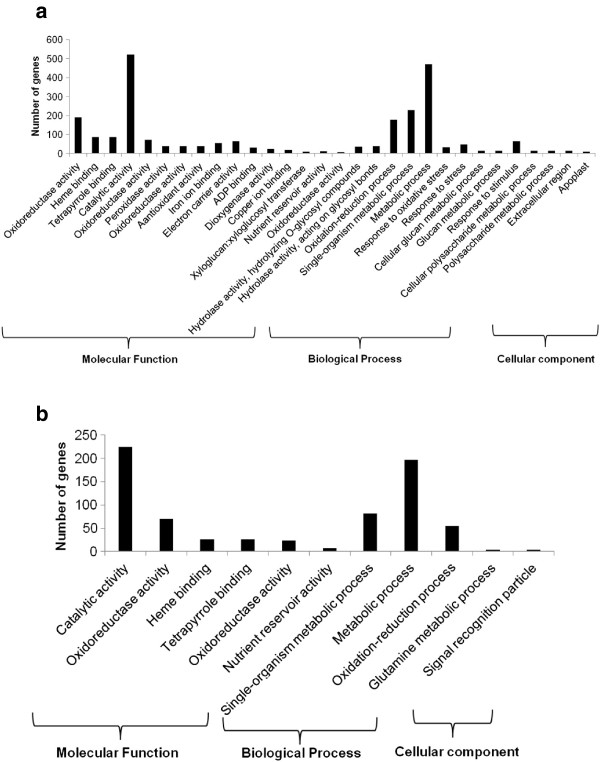
**Histogram showing Gene Ontology functional enrichment analysis. a)** GO terms for DEG transcripts abundant in sensitive genotypes. **b)** GO terms for DEG transcripts abundant in tolerant genotypes.

After identifying DEG transcripts from 12 pair-wise comparisons, we separated the DEGs abundant in sensitive genotypes from the DEGs abundant in tolerant genotypes. The functional annotations of DEG transcripts were established using GO::TermFinder to see which GO terms are enriched in these two groups of genotypes. GO analysis classify the gene transcripts and gene products into their corresponding biological processes (BP), molecular function (MF), and cellular component (CC). The DEG transcripts with known GO annotation were categorized in to 30 functional groups in sensitive genotypes (Figure 2a) and 11 groups in tolerant genotypes (Figure 2b). In the molecular function ontology, the DEG transcripts associated with catalytic activity were the most abundant group in both sensitive (522) and tolerant (225) genotypes. DEG transcripts associated with heme, tetrapyrrole binding, and nutrient reservoir activity encoding storage proteins such as albumins were found common between sensitive and tolerant genotypes. GO terms associated with molecular functions like peroxidase, hydrolase, antioxidant, dioxigenase, electron carrier activity are enriched in sensitive genotypes. In the biological processes ontology, GO terms associated with metabolic process were the most enriched in sensitive (471) and tolerant (197) genotypes. DEG transcripts related to stress responses including oxidative stress and stimuli were found in sensitive genotypes. Three DEGs associated glutamine metabolic process GO terms were enriched in tolerant genotypes. With respect to cellular component ontology, DEG transcripts associated with extracellular region and apoplast GO terms were found in sensitive genotypes and signal recognition particle terms in tolerant genotypes.

### Functional enrichment of significant genes

In addition to GO analysis, the DEG transcripts abundant in sensitive genotypes and tolerant genotypes were mapped to terms in KEGG database, and compared with the sorghum reference genome to identify significantly enriched metabolic or signal transduction pathways. DEG transcripts with KEGG annotation were categorized into 78 pathways in sensitive genotypes (Additional file 6A) and 68 pathways in tolerant genotypes (Additional file 6B). In both sensitive and tolerant genotypes 59 pathways were common, including inositol phosphate, pyruvate, starch and sucrose, fructose and mannose metabolism, and citrate (TCA) cycle. DEGs associated with flavonoids, stilbene and lignin biosynthesis, fluorine degradation, gamma-hexachlorocyclohexane degradation, ascorbate and aldarate metabolism were enriched in all genotypes. The amino acid biosynthetic pathways (phenylalanine, tyrosine, and tryptophan) and primary metabolism pathways like fatty acid, nitrogen, amino sugars, vitamin B6, galactose, glutathione, sulphur and riboflavin were significantly enriched in both sensitive and tolerant genotypes. DEG transcripts associated with alanine and aspartate metabolism, pentose phosphate pathway, amino sugars and thiamine metabolism, aromatic amino acids (phenyl alanine, tyrosine and tryptophan), sterol and alkaloid biosynthetic pathways are enriched in sensitive genotypes (Additional file 6A). The pathways related to folate, pentothenate, aminoacyl-tRNA, lysine biosynthesis and branched chain amino acids (valine, leucine and isoleucine) degradation pathways were enriched in tolerant genotypes. In addition, DEGs related to histidine, aminophosphonate and methionine metabolisms were also enriched in tolerant genotypes (Additional file 6b).

### Comparison of DEGs between N-stress tolerant and sensitive genotypes

**Figure 3:**
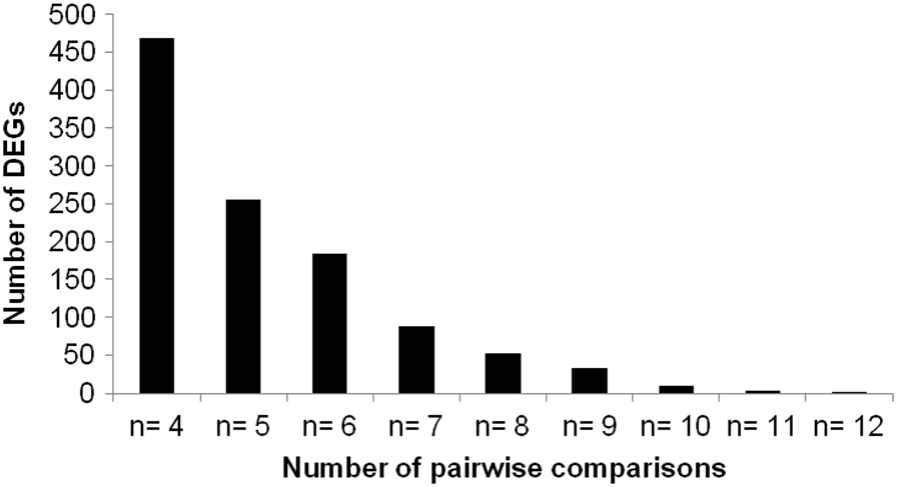
Number of DEGs among 12 pair-wise comparisons made between three sensitive and four tolerant genotypes; n = the number of comparisons in which the given gene transcript was differentially expressed.

The number of DEG transcripts between low-N tolerant and sensitive genotypes of sorghum were calculated for n = 4 through n = 12, where n is the number of pair-wise comparisons in which the given gene transcript was differentially expressed. Only two transcripts showed differential expression in all 12 pair-wise comparisons made between all three low-N tolerant and four sensitive genotypes. A total of 183 genes showed differential expression when n = 6, while 33 genes showed differential expression when n = 9 (Figure 3). From these DEGs, transcripts that showed differential expression among the four tolerant genotypes (Additional file 3) as well as among three sensitive genotypes (Additional file 4) were discarded. This process would differentiate the DEG transcripts involved in low-N tolerance from the genes differentially expressed due to unrelated genotype differences. A total of 115 DEG transcripts when n = 6 (Additional file 7) were found common between four tolerant and three sensitive genotypes. Of these, 88 were abundant in sensitive genotypes and 27 DEG transcripts were abundant in tolerant genotypes.

### Differential expression of nitrogen metabolism genes in sorghum genotypes

RNA-seq results for known nitrogen transport and assimilation genes indicate that N-stress increased the abundance of gene transcripts encoding high affinity nitrate transporters in tolerant genotypes (Table 4). For example, transcript encoding nitrate transporter NRT2.5 or NRT2.7 was abundant in tolerant genotypes compared to CK60. Other transcripts encoding nitrate transporters, NRT2.2, NRT2.3, and NRT2.6 were also abundant in China17 and KS78 compared to sensitive genotypes. Fd NADP (+) reductase (FNR) transcript was abundant in China17 compared to sensitive genotypes, but the response was slight. Expression of a transcript encoding enzymes involved in ammonia assimilation, glutamate synthetase (GOGAT-3) was abundant in KS78 and high-NUE bulk, but not in China17 and San Chi San relative to CK60. Conversely, glutamine synthetase (GS-2) transcript was abundant in CK60 compared to tolerant genotypes. Similarly, transcripts encoding genes involved in nitrate assimilation, nitrate reductase (NR-1), nitrite reductase (NiR) were abundant in CK60 and BTx623 compared to the high-NUE bulk.

**Table 4:** Differential expression of nitrogen metabolism genes among sorghum genotypes

**Gene name**	**Locus**	**Log(FC) = log**_**2 **_**(sensitive/tolerant genotype)**											
		**1/3**	**1/4**	**1/5**	**1/6**	**2/3**	**2/4**	**2/5**	**2/6**	**7/3**	**7/4**	**7/5**	**7/6**
NRT2.5 or 2.7	Sb03g032310	-1.6	-3.4	-4.1	-2	ns	ns	-1.9	ns	ns	-1.6	-2	ns
HANT:NRT2.2	Sb04g001000	ns	-1.9	-2.2	ns	ns	-1.6	-1.9	ns	ns	-1.8	-2.2	ns
HANT:NRT2.3	Sb04g001000	ns	-1.9	-2.2	ns	ns	-1.6	-1.9	ns	ns	-1.8	-2.2	ns
HANT:NRT2.6	Sb04g001000	ns	-1.9	-2.2	ns	ns	-1.6	-1.9	ns	ns	-1.8	-2.2	ns
GOGAT-3	Sb03g031310	ns	ns	-1.8	-1.8	ns	ns	ns	ns	-2	ns	ns	ns
NiR	Sb04g034160	ns	ns	ns	3.6	ns	ns	ns	3.9	-1.6	-1.5	ns	2.1
NR-1	Sb07g022750	ns	ns	ns	3.7	ns	2.1	ns	4.8	-1.9	ns	-1.9	1.8
GS-2	Sb06g031460	ns	4.1	5.3	6	-4	ns	ns	ns	-2.6	ns	ns	ns
FNR	Sb01g006100	ns	ns	ns	ns	ns	-1.6	ns	ns	ns	ns	ns	ns

ns = non-significant when FDR ≤ 1; Log(FC) is the log_2_ ratio of gene transcript between sensitive genotypes [CK60 (1), BTx623 (2), low-NUE bulk (7)] and tolerant genotypes (San Chi San (3), China17 (4), KS78 (5), High-NUE bulk (6)]; If Log(FC) <0, negative values indicate transcript is abundant in tolerant genotypes and if Log(FC) >0, positive values indicate transcript is abundant in sensitive genotypes.

### DEG transcripts abundant in sensitive genotypes under N-stress

A higher number of gene transcripts were abundant in sensitive genotypes under N-stress, (Additional file 7), some of which were listed in Table 5. Transcripts encoding flavonoids and stilbene biosynthesis including chalcone and stilbene synthase, flavanone 3-hydroxylase, choline monooxygenase and flavin containing domains were abundant in sensitive genotypes. Abundance of a DEG transcript encoding leucoanthocyanidin dioxygenase, and transcripts encoding cytochrome P450’s (CYP71A25, CYP87A2, CYP72A15) were higher in CK60 and BTx623 compared to San Chi San, China17 and KS78. The transcripts encoding genes involved in cell wall modification including beta-expansin, alpha/beta hydrolases, peroxidases, chitinase A glycosyl hydrolase and beta-1, 3-glucanase had higher abundance in sensitive genotypes. N-stress increased the abundance of gene transcripts related to phytohormones such as auxins, and cytokinins in sensitive genotypes (Table 5). The transcript abundance of regulatory genes, such as transcription factors and protein kinases, was also differential between the genotypes. Here, five kinases showed higher abundance in sensitive genotypes, including cysteine-rich receptor like kinase (CRK55), PR5-like pathogen resistance receptor kinase (ARK1AS), S-locus lectin protein kinase, PEP1 receptor kinases. Several transcription factors also showed higher abundance in sensitive genotypes including a putative MYB transcription factor and auxin responsive transcription regulators (ARF2, OsSAUR4).

**Table 5:** List of DEG transcripts abundant in sensitive genotypes

		**Log(FC) = log**_**2 **_**(sensitive/tolerant genotype)**											
**Gene annotation**	**Gene id**	**1/3**	**1/4**	**1/5**	**1/6**	**2/3**	**2/4**	**2/5**	**2/6**	**7/3**	**7/4**	**7/5**	**7/6**
**Flavanoids and stilbene biosynthesis**													
Chalcone and stilbene synthase	Sb07g004700	3.9	5.0	8.4	3.4	3.1	4.2	7.5	2.6	**	**	6.8	**
Choline monooxygenase	Sb10g028700	5.0	6.0	7.1	**	4.2	5.2	6.4	**	4.4	5.5	6.6	**
Polyamine oxidase 1, Flavin-containing domain	Sb07g005780	3.9	4.2	2.6	1.9	3.2	3.5	1.9	**	3.4	3.7	2.0	1.3
Leucoanthocyanidin dioxygenase	Sb06g014550	3.3	3.0	**	2.9	2.8	2.5	**	2.4	2.2	1.9	**	1.8
Anthocyanidin 5,3-O-glucosyltransferase	Sb09g026280	**	**	3.2	3.7	**	**	2.5	2.9	**	2.4	2.8	3.3
CYP71A25 cytochrome P450,	Sb07g000550	11.8	7.5	4.8	4.9	8.7	4.5	1.7	1.8	10.2	5.9	3.1	3.2
CYP87A2 cytochrome P450,	Sb05g004900	7.6	**	7.7	3.5	6.6	**	6.6	2.4	6.0	**	6.1	**
Glutathione S-transferase	Sb04g003850	9.3	9.2	7.0	**	9.6	9.5	7.3	1.9	9.1	9.1	6.8	**
**Cell wall metabolism**													
Beta-1,3-glucanase	Sb08g019670	3.9	3.9	**	3.3	2.1	2.2	**	**	2.2	2.2	**	**
Beta-expansin	Sb10g028070	2.8	3.9	**	2.6	**	2.6	**	**	2.3	3.5	**	2.1
Peroxin 13	Sb02g003110	2.5	2.2	**	**	2.5	2.2	**	1.6	2.0	1.7	**	**
Peroxidase superfamily protein	Sb09g020980	9.8	**	**	4.0	8.4	**	**	2.6	8.5	**	**	2.6
Peroxidase superfamily protein	Sb09g021000	3.8	**	**	3.4	2.5	**	**	2.1	2.6	**	**	2.2
Alpha/beta-Hydrolases	Sb10g021250	10.1	10.0	**	3.5	8.8	8.7	**	2.2	9.0	8.9	**	2.4
Alpha/beta-Hydrolases	Sb1306s002010	7.2	7.9	**	3.8	6.3	7.0	**	2.9	6.0	6.7	**	2.6
Glycosyl hydrolases family 17	Sb08g019670	3.9	3.9	**	3.3	2.1	2.2	**	**	2.2	2.2	**	**
Chitinase A glycosyl hydrolase	Sb05g023710	2.7	**	**	1.9	2.3	**	**	1.5	2.3	**	**	1.5
**Phytohormones**													
SAUR-like auxin-responsive protein	Sb06g001800	**	3.5	**	3.6	**	**	**	3.4	3.6	3.5	**	3.5
Auxin response factor 2 (ARF 2)	Sb06g011767	6.5	6.5	**	**	7.3	7.3	**	2.2	7.8	7.7	**	2.6
Auxin-induced protein 5NG4	Sb04g000450	3.8	5.5	**	8.0	3.2	4.8	**	7.3	**	**	**	**
Cytokinin-O-glucosyltransferase 2	Sb06g018490	12.4	7.5	**	2.9	12.4	7.6	**	2.9	12.1	7.2	**	2.5
**Kinases**													
CRK55, Cystein rich RLK 55	Sb01g039360	**	2.2	2.1	**	**	1.3	1.2	**	2.0	2.3	2.2	**
PR5-like receptor kinase	Sb03g025630	**	4.1	3.5	**	**	3.7	3.0	**	**	4.7	4.0	**
Protein kinase superfamily protein	Sb01g041910	3.2	**	**	3.2	3.2	**	**	3.1	2.4	**	**	2.4
S-locus lectin protein kinase	Sb09g001750	**	**	5.9	**	2.9	3.6	7.3	**	**	2.3	5.9	**
PEP1 receptor-1 kinase	Sb07g021940	3.0	4.1	**	**	2.9	4.0	**	1.8	1.9	3.1	**	**
**Transcription factors**													
MYB-like transcription factor	Sb09g030390	2.9	4.2	**	2.4	1.7	2.9	**	**	**	2.4	**	**
AP2/B3-like transcriptional factor	Sb01g014400	4.4	4.2	2.5	**	6.0	5.7	4.1	1.8	3.2	2.9	**	**
Auxin response factor 2	Sb06g011767	6.5	6.5	**	**	7.3	7.3	**	2.2	7.8	7.7	**	2.6
SAUR-like auxin-responsive protein	Sb06g001800	**	3.5	**	3.6	**	**	**	3.4	3.6	3.5	**	3.5

The transcriptional abundance of DEGs from 12 pair-wise comparisons (1/3, 1/4, 1/5, 1/6, 2/3, 2/4, 2/5, 2/6, 7/3, 7/4, 7/5, and 7/6) made between three sensitive genotypes [CK60 (1), BTx623 (2) and the low-NUE bulk (7)] with each of the four with each of the four tolerant genotypes [San Chi San (3), China17 (4), KS78 (5) and the high-NUE bulk (6)] were summarized. **Not significant when FDR ≤ 0.001; Log(FC) is the log_2_ ratio of gene transcript between sensitive and tolerant genotypes; If Log(FC) >0, positive values indicate transcript is abundant in sensitive genotypes.

### DEG transcripts abundant in tolerant genotypes under N-stress

In this study, 27 gene transcripts showed higher abundance in tolerant genotypes compared to sensitive genotypes under N-stress conditions. These transcripts encoded genes involved in membrane transporter, defense, protein synthesis and protein turnover (Table 6). Genes involved in membrane transport include, a lysine histidine transporter 1(LHT1), whose expression was abundant in San Chi San, China17 and high-NUE bulk compared to sensitive genotypes under N-stress. A transcript encoding SEC14 cytosolic factor family protein was also abundant in San Chi San, China17 and KS78 relative to CK60 and BTx623. The abundance of a gene transcript encoding a protein with ankyrin repeat was higher in tolerant genotypes relative to CK60. The transcripts encoding many ribosomal genes involved in protein synthesis including structural constituent of ribosome L16p/L10 and translation elongation factors (Tu), were abundant in tolerant genotypes compared to sensitive genotypes. In addition, transcripts encoding genes involved in abiotic stress response, like drought induced family protein were abundant. Genes involved in detoxification of xenobiotics like UDP-Glycosyltransferase and Glutathione-S-transferase were abundant in tolerant genotypes.

**Table 6:** List of DEG transcripts abundant in tolerant genotypes

		**Log(FC) = log**_**2 **_**(sensitive/tolerant genotype)**											
**Gene annotation**	**Gene id**	**1/3**	**1/4**	**1/5**	**1/6**	**2/3**	**2/4**	**2/5**	**2/6**	**7/3**	**7/4**	**7/5**	**7/6**
Ankyrin repeat	Sb07g002190	-7.5	-8.4	-7.7	-6.9	**	**	**	**	**	-3.0	-2.2	**
LHT1 lysine histidine transporter 1	Sb01g038720	-7.0	-7.7	**	-6.7	-7.0	-7.6	**	-6.7	**	**	**	**
SEC14 cytosolic factor	Sb05g026380	-7.8	-6.4	-7.7	**	-3.7	**	-3.6	**	**	**	**	**
Drought induced 19 protein	Sb04g013790	-3.8	-4.6	**	-3.2	-2.3	-3.1	**	-1.7	**	-2.3	**	**
Translation elf- Tu	Sb02g007166	-4.7	-4.6	**	-4.5	-4.1	-4.0	**	-3.9	-2.4	-2.3	**	**
BTB-POZ and MATH domain 1	Sb07g026735	-5.1	-5.6	**	**	-7.3	-7.8	**	-6.5	**	-2.3	**	**
Ribosomal protein (L16p/L10e)	Sb01g036330	-2.7	-3.4	-2.3	-2.0	**	-1.7	**	**	**	-1.4	**	**
Glutathione S-transferase	Sb09g003700	-2.1	-2.6	**	**	-2.4	-3.0	**	-1.3	-1.9	-2.5	**	**
Phosphatases	Sb08g019110	-2.1	-2.9	-2.1	-2.0	-2.9	-3.6	-2.9	-2.8	**	-1.6	**	**
Leucine-rich receptor-like kinase	Sb04g003840	-2.7	-2.6	-2.7	**	-1.9	-1.8	-1.9	**	**	**	-1.5	**
Phosphoglycerate mutase	Sb06g000380	-3.4	-3.3	**	-3.6	**	-1.5	**	-1.7	**	**	**	-1.3
RmlC-like cupins	Sb01g019830	-3.5	-5.0	-3.2	-3.2	**	-2.1	**	**	**	-2.0	**	**
Homeobox associated leucine zipper	Sb07g029150	-3.6	-2.7	**	**	-4.2	-3.4	-3.1	-2.8	**	**	**	**
Expressed protein	Sb08g019270	-3.7	-3.7	**	-3.0	-3.9	-4.0	**	-3.3	**	**	**	**
Transducin	Sb04g022100	-3.8	-3.6	**	-3.3	-3.2	-2.9	**	-2.6	**	**	**	**
Expressed protein	Sb04g000700	-3.9	-3.9	**	**	-6.9	-6.9	**	**	-3.5	-3.5	**	**
Trypsin family protein with PDZ domain	Sb08g015916	-4.3	**	**	-4.9	-4.2	**	**	-4.8	-3.1	**	**	-3.7
3-oxo-5-alpha-steroid 4-dehydrogenase	Sb02g003510	-4.9	-4.5	**	-3.2	-3.2	-2.8	**	-1.6	-2.8	-2.4	**	-1.1
F-box domain containing protein	Sb02g001640	-5.4	**	**	-4.4	-5.3	**	**	-4.3	-3.5	**	**	-2.4
DNA binding transposon protein	Sb05g020750	-7.0	-7.4	**	-7.4	-7.0	-7.3	**	-7.3	**	-2.6	**	**
Expressed protein	Sb04g000690	-7.7	-7.9	**	-7.0	-7.6	-7.9	**	-7.0	-5.5	-5.8	**	-4.9
Leucine Rich Repeat family protein	Sb06g001645	-7.9	-7.6	**	-7.2	-7.9	-7.5	**	-7.1	**	**	**	**
Expressed protein	Sb04g012640	-8.3	**	**	-8.2	-6.0	**	**	-5.9	-3.9	**	**	-3.7
Cell wall invertase 2	Sb0067s002240	-9.1	-7.4	**	-7.1	-6.7	-5.1	**	-4.8	-3.5	**	**	**
Hypothetical protein	Sb04g012541	-9.1	**	**	-9.3	-9.0	**	**	-9.3	-4.2	**	**	-4.5
Cupin domain containing protein	Sb07g005307	**	-2.9	**	**	-7.7	-9.2	-8.0	-7.9	**	-2.7	**	**
UDP-Glycosyltransferase	Sb04g027470	**	**	**	**	-6.2	-4.5	-5.2	-4.3	-2.3	**	-1.2	**

The transcriptional abundance of DEGs from 12 pair-wise comparisons (1/3, 1/4, 1/5, 1/6, 2/3, 2/4, 2/5, 2/6, 7/3, 7/4, 7/5, and 7/6) made between three sensitive genotypes [CK60 (1), BTx623 (2) and the low-NUE bulk (7)] with each of the four tolerant genotypes [San Chi San (3), China17 (4), KS78 (5) and the high-NUE bulk (6)] were summarized. **Not significant when FDR ≤ 0.001; Log(FC) is the log_2_ ratio of gene transcript between sensitive and tolerant genotypes; If Log(FC) <0, negative values indicate transcript is abundant in tolerant genotypes.

## Discussion

The focus of our study is to identify common genes that are differentially expressed between low-N tolerant and sensitive genotypes having different genetic backgrounds with differential response to N-stress. To select the genotypes with differential response to N, five sorghum genotypes (CK60, BTx623, San Chi San, China17 and KS78) and RILs from CK60 × San Chi San were evaluated under field conditions provided with full N (100 Kg ha^-1^ fertilizer) and N-stress (0 Kg ha^-1^). The phenotypes of five sorghum genotypes, five best and worst performing RILs tested under contrasting N-regimes showed that the mean values of plant height, biomass and grain yields were reduced from NN to LN field conditions (Table 1). Under controlled conditions, the average weights of roots and shoots of three week-old seedlings were also reduced from full N (100% Hoagland solution) to N-stress (Table 2). In maize, a 38% reduction in grain yield was observed from high-N to low-N conditions [37], which likely results from limitation of photosynthetic output caused by lower production of proteins like Ribisco [17]. Under N-stress conditions, the lower root and shoot weights of three week old seedlings and lower biomass and grain yields of CK60, BTx623 and RILs 1-5 from field conditions, indicates their sensitivity to the limited N. San Chi San, China17 and RILs 6-10 grow taller and have higher biomass and grain yields in the field conditions and had higher root and shoot weights in the seedling stage, indicating their greater tolerance to the limited N. The RILs showed transgressive segregation and this suggested a polygenic inheritance of the traits. Maranville and Madhavan [28] showed that assimilation efficiency indices were significantly greater for the tolerant Chinese lines (San Chi San and China17) compared to sensitive US-lines (CK60 and BTx623) at both low and high N levels and the Chinese lines retained greater phosphoenolpyruvate carboxylase (PEPcase) activity under N-stress. This suggests that PEPcase and enzymes associated with PEP synthesis are perhaps responsible for maintaining relatively high photosynthesis under N-stress, and resulted in greater biomass accumulation of the tolerant genotypes [28].

### Comparison of transcriptomes between sorghum genotypes

To identify common DEGs between genotypes having differential response to N-stress, RNA-seq was used to compare the transcriptomes of root tissues of genotypes grown under N-stress. From RNA-seq data, a total of 12 pair-wise comparisons were made by comparing three sensitive genotypes with each of the four tolerant genotypes to find common DEG transcripts across all genotypes. In order to differentiate non-specific DEG transcripts from those related to N-stress, the transcripts between four tolerant genotypes and three sensitive genotypes were inter-compared one by one. The transcripts that showed differential expression among tolerant (Additional file 3) and sensitive (Additional file 4) genotypes were discarded from the list of DEGs between 12 pair-wise comparisons. A total of 115 common DEG transcripts were observed between three sensitive and four tolerant genotypes, which could be related to N-stress (Additional file 7).

Expression analysis using qRT-PCR of selected genes confirmed their differential expression under low-N conditions (Additional file 5b). Furthermore, the differential expression of these genes was either absent, reduced or even reversed when plants were grown under full-N conditions (Additional file 5c). This is consistent with the suggestion that the selected genes are differentially expressed as a specific response to N-deficiency.

### Differential expression of known nitrogen metabolism genes in sorghum genotypes

In general, N-starvation increases the expression of high-affinity transport systems for nitrate and ammonium [7]. Here, N-stress increased the abundance of high affinity nitrate transporter gene transcripts (NRT2.5 or NRT2.7, NRT2.2, NRT2.3, and NRT2.6) in tolerant genotypes one to four-fold relative to sensitive genotypes (Table 4). Earlier reports showed that high affinity nitrate transporters were expressed in N-starved seedlings of *Arabidopsis*[38,39]. In rice, the nitrate transporter (OsNRT2.2) in association with OsNAR2.1 transports nitrate in the high affinity concentration range in roots [40]. The increased nitrate could promote the elongation of lateral roots [5]. Conversely, the abundance of nitrate assimilatory gene transcripts, NR-1 and NiR, and ammonia assimilatory gene, GS-2 was higher in sensitive genotypes. GS-2 transcript increased in CK60 compared to China17, KS78 and the high-NUE bulk. However, San Chi San had higher levels of GS-2 transcript compared to BTx623 and low-NUE bulk, indicating a lack of functional redundancy in the expression of gene transcripts. The nitrate assimilation genes and GS-2 could be highly expressed to sustain the stress conditions. Overall, known nitrate transporter and assimilation genes showed very little change in expression between the tolerant and sensitive genotypes, indicating that the expression of basic N metabolism genes may be genotype independent. In the analysis of gene expression profile comparisons of rice using microarray, Lian et al [23] observed similar results; genes involved in N uptake and assimilation showed little response to N-stress.

### Abundance of transcripts in sensitive genotypes under N-stress

DEG transcripts associated with secondary metabolism like flavonoids and anthocyanin biosynthesis, as well as those associated with abiotic stress responses, were abundant in sensitive genotypes (Table 5). Such expression changes may be involved in the plant’s tolerance to N-stress. The role flavonoids play in the sensitive genotypes under N-stress is not known. However, expression of flavonoid biosynthetic pathway genes was also reported in soybean [26] and *Arabidopsis*[30] when genotypes grown under severe N-stress. In addition, the transcripts encoding Cytochrome P450s were abundant in sensitive genotypes (Table 5). Cytochrome P450s catalyze oxidation of a wide range of chemical reactions by activating dioxygen [41] and were reported to play an important role in biosynthesis of anthocyanin’s in response to stress [42]. Similarly, four Cytochrome P450s were expressed higher in rice seedlings under N-stress [43].

A transcript encoding putative MYB transcription factor was abundant in sensitive genotypes (Table 5). It was reported that MYB genes contribute to the control of flavonoid biosynthesis in a wide range of plant species (maize, petunia) often in combination with other regulatory genes [44]. A DEG transcript encoding choline monooxygenase gene, an iron sulphur enzyme involved in synthesis of glycine betaine in plants [45], was abundant in low-N sensitive genotypes CK60 and BTx623. It was reported that many species (maize, soybean, rice, and wheat) of transgenic plants with its over-expression had significantly increased glycine betaine content. Glycine betaine is a nitrogenous compound and acts as an osmoprotectant and its accumulation was associated with abiotic stress tolerance [46]. In addition, transcript encoding Glutathione-S-transferase (GST) was also abundant in sensitive genotypes. GST catalyzes the glutathione-dependent detoxification reactions and the reduction of hydroperoxides. GSTs may act as binding proteins that sequestrate flavonoids in the vacuole for protection against environmental stresses [47]. Therefore, induction of the flavonoid pathway may be a characteristic response of genotypes sensitive to N-stress.

Alteration in the lipid composition of plant cell membranes is one of the multiple defense strategies [48]. Here, the transcripts encoding genes involved cell wall modification like peroxidases, peroxin-13, hydrolases like glycosyl hydrolase 17, were abundant in sensitive genotypes CK60 and BTx623. These proteins may be important for wall assembly, remodeling during growth, development and stress responses. Since nitrogen stress causes reduction in cell growth, it was not surprising to find abundance of a β-expansin gene transcript. Expansins play important roles in root growth and development under nutrient and abiotic stress conditions and are also involved in cell wall expansion [49,50]. Therefore, the sensitive genotypes defend the stress and maintain the growth by altering the cell wall.

Phytohormones such as auxins and cytokinins were also reported to play important roles during the adaptation to limited N [51]. The transcripts encoding auxin response factors (SAUR-like, ARF2) and auxin inducible proteins, 5NG4, were abundant in CK60 and BTx623 compared to tolerant genotypes (Table 5) under stress. Earlier reports showed that inhibition of auxin transport resulted in increased levels of MtN21-like-a/b and 5NG4 [52], led to localized increase in auxin concentration through a blockage of the PIN1 cycling [53], and resulted in reduced number of emerging lateral roots. The abundance of transcripts encoding auxin inducible proteins in sensitive genotypes could have resulted in their reduced root mass under N-stress (Table 2).

Kinases play important roles in the development of eukaryotic cells, such as cell cycle control and cell-type determination and differentiation [54]. Kinases help the organism to cope with changing conditions and stresses in the environment. Because some of their targets are transcription factors, they also play a role in regulating transcription [55]. In this study, DEG transcripts encoding five kinases were abundant in sensitive genotypes, which include cysteine-rich receptor like kinases (Table 5). Previous research indicated that receptor-like kinases play important roles in plant growth and development [56] and had differential expression in soybean genotypes grown under N-stress [26]. Therefore, we hypothesize that these kinases might be important for adaptation to N-stress in sensitive genotypes of sorghum.

### Abundance of transcripts in tolerant genotypes under N-stress

Under N-stress, plants tend to increase their N uptake ability by regulating physiological, biochemical activities and by changing root morphology including increased root length, root hair density and lateral root number [57]. We found that tolerant genotypes adapt to N deficiency by producing higher root mass compared to sensitive genotypes (Table 2). Also, many gene transcripts involved in nitrate transport (Table 4) were present at higher levels in tolerant genotypes. It is proposed that N-metabolism related gene transcripts especially those encoding transporters, were increased in tolerant genotypes in order to uptake nitrate or amino acids from soil more efficiently and to produce more nitrogen containing metabolites required for their survival under N-stress.

The soil contains significant amounts of organic nitrogen derived from decomposition of organic matter by microorganisms, which is rich in amino acids. Plants have different capacities to take up these amino acids through putative amino acid transporters localized on the root epidermal cells [58]. In this study, a DEG transcript encoding high affinity amino acid transporter, LYSINE HISTIDINE TRANSPORTER1 (LHT1), was massively expressed in San Chi San and China17 compared to sensitive genotypes (Table 6). It was reported that being expressed in the root, LHT1 is responsible for uptake of amino acids from soil into root tissue [59], and distributes from roots to shoots through xylem [60] for further metabolism especially under N-limited conditions. The amino acid uptake, and thus nitrogen use efficiency of the tolerant genotypes, could be higher with increased LHT1 expression under limited inorganic N supply.

To survive under N-stress, some genes involved in alleviating the detrimental effect of stress are abundantly expressed, which could facilitate tolerance to the stress. In this study, cell wall invertase-2 (CWINV2) transcript was massively increased in San Chi San and China17 (Table 6), indicating that sucrose degradation was increased in tolerant genotypes. A similar observation was made in the leaves of a water stress resistant cultivar of wheat [61]. It is believed that the enhanced invertase expression in the roots of tolerant genotypes may contribute to the rapid cycling of sucrose, thus promoting carbon partitioning in favor of sucrose accumulation for counteracting the stress condition [20]. In addition, the transcript of SEC14 cytosolic factor family protein was abundantly expressed in tolerant genotypes compared to CK60 and BTx623 (Table 6). It is also known as phosphatidylinositol/phosphatidylcholine transfer protein, and is located in the Golgi membrane. There, it acts as a signal precursor and activates stress responsive genes, phospholipids and galactolipids [62], which increase the membrane stability and provides stress tolerance [63]. Gene transcripts responsible for numerous cellular activities, including protein biosynthesis, modification, and degradation enzymes were abundantly expressed in tolerant genotypes. Transcripts encoding ribosomal genes involved in protein biosynthesis, including structural constituent of ribosome L16p/L10 and translation elongation factors (EF1A) were also abundant in tolerant genotypes (Table 6).

## Conclusion

Identification of common DEG transcripts between sorghum genotypes with contrasting stress tolerance would facilitate a better understanding of the genetic bases of low-N tolerance. Here, Illumina RNA-seq analysis demonstrated that gene transcripts involved in abiotic stress response, and secondary metabolism were abundantly expressed in sensitive genotypes of sorghum under N-stress. Higher expression of these gene transcripts could enable the sensitive genotypes to thrive under stress conditions.

The magnitude of expression changes in N transporter, assimilation genes between tolerant and sensitive genotypes was less. Conversely, many genes not directly involved in nitrate metabolism had differential expression under N-stress. In addition, the magnitude of change in the expression of these genes was different between the genotypes with varying degrees of tolerance to N-stress. While sorghum seems to have a typical nitrate metabolism process, it appears that many genes indirectly involved in nitrate metabolism that respond to an nitrogen stress treatment, are important for the observed differences between tolerant and sensitive genotypes of sorghum. The DEG transcripts found between sensitive and tolerant genotypes of sorghum in this study should provide useful information for understanding how different sorghum genotypes encounter the N-stress at seedling stage and how tolerant and sensitive genotypes can adapt to N-stress conditions. Furthermore, the transcriptomes of stress tolerant and sensitive genotypes grown under full nitrogen were evaluated, suggested that the selected genes were differentially expressed as a specific response to N-deficiency. The DEGs from tolerant genotypes would be the potential candidates to study further for improving NUE of sorghum and related crop plants.

### Availability of supporting data

We deposited the RNA-seq data in Gene Expression Omnibus (GEO; http://www.ncbi.nlm.nih.gov/projects/geo/) repository with an accession number GSE54705.

## Competing interests

The authors declare that they have no competing interests.

## Author’s contributions

MG designed the study, managed growing the plants, isolated RNA for RNA-seq, analyzed data, performed qRT-PCR, wrote and revised the manuscript, YD and CZ analyzed the RNA-seq data, ARK helped in the field experiments, analyzed data, drafted and revised the manuscript related to biochemical pathways; DRH designed the study and co-wrote the manuscript; ID coordinated the project and critically reviewed the manuscript. All the authors read and approved the final manuscript.

## Supplementary Material

Additional file 1 Oligonucleotide sequences used for Real-Time RT-PCR (qRT-PCR). Click here for file

Additional file 2 List of all DEGs among 12 pair-wise comparisons (1/3, 1/4, 1/5, 1/6, 2/3, 2/4, 2/5, 2/6, 7/3, 7/4, 7/5, and 7/6) made between three sensitive [CK60 (1), BTx623 (2), and low-NUE RIL bulk (7)] and four tolerant [San Chi San (3), China17 (4), KS78 (5) and high-NUE RIL bulk (6)] genotypes of sorghum. Click here for file

Additional file 3 List of DEGs among four tolerant genotypes [San Chi San (3), China17 (4), KS78 (5) and high-NUE RIL bulk (6)]. Click here for file

Additional file 4 List of DEGs among three sensitive genotypes [CK60 (1), BTx623 (2), and low-NUE RIL bulk (7)]. Click here for file

Additional file 5 **Confirmation of differentially expressed candidate genes. A)** The Fold changes observed between 12 pair-wise comparisons (1/3, 1/4, 1/5, 1/6, 2/3, 2/4, 2/5, 2/6, 7 /3, 7/4, 7/5, and 7/6) made between three sensitive genotypes [CK60 (1), BTx623 (2) and the low-NUE bulk (7)] with each of the four tolerant genotypes [San Chi San (3), China17 (4), KS78 (5) and the high-NUE bulk (6)] in RNA-seq. **B)** Confirmation of the fold changes observed in RNA-seq with Real-Time qRT-PCR for the cDNA’s extracted from root tissues of seedling grown under N-stress. **C)** Checking the expression levels of candidate genes for the cDNA’s extracted from root tissues of seedlings grown under full N (100% Hoagland solution). Click here for file

Additional file 6 **Pathway enrichment analysis of common DEG transcripts. A.** Pathways associated with DEG transcripts abundant in sensitive genotypes. **B.** Pathways associated with DEG transcripts abundant in tolerant genotypes. Click here for file

Additional file 7 List of common DEGs among 12 pair-wise comparisons (1/3, 1/4, 1/5, 1/6, 2/3, 2/4, 2/5, 2/6, 7/3, 7/4, 7/5, and 7/6) made between three sensitive [CK60 (1), BTx623 (2), and low-NUE RIL bulk (7)] and four tolerant [San Chi San (3), China17 (4), KS78 (5) and high-NUE RIL bulk (6)] genotypes of sorghum. Click here for file
